# Comparative intra- and inter-observer reliability of two methods for evaluating intraoperative ultrasonography-based spinal cord hyperechogenicity intensity in degenerative cervical myelopathy

**DOI:** 10.1186/s12891-022-05517-0

**Published:** 2022-07-02

**Authors:** Huachuan Wu, Guoliang Chen, Xianlong Li, Zhengya Zhu, Zuofeng Xu, Xizhe Liu, Shaoyu Liu

**Affiliations:** 1 Department of Orthopaedic Surgery, Innovation Platform of Regeneration and Repair of Spinal Cord and Nerve Injury, The Seventh Affiliated Hospital, Sun Yat-Sen University, Shenzhen, 518107 China; 2 Orthopaedic Research Institute/Department of Spinal Surgery, Guangdong Provincial Key Laboratory of Orthopedics and Traumatology, The First Affiliated Hospital of Sun Yat-Sen University, Guangzhou, 510080 China; 3 Department of Ultrasound, The Seventh Affiliated Hospital of Sun Yat-Sen University, Shenzhen, China

**Keywords:** Degenerative cervical myelopathy, Cervical spondylosis, Intraoperative ultrasound, Ultrasound imaging, Spinal cord, Intra- and interobserver reliabilities

## Abstract

**Objectives:**

During French-door laminoplasty, a linear array transducer of IOUS was used to observe and record the spinal cord decompression. To acquire a higher-reliability method, and compare the in-observer and inter-observer reliability of two methods in evaluating the hyperechoic intensity of spinal cord ultrasound in degenerative cervical myelopathy (DCM).

**Background:**

The intensity of spinal cord hyperechogenicity is considered as a potential predictor of neurological recovery in DCM after decompression, but the accuracy of gray value ratio (GVR) is affected by many factors.

**Methods:**

Totally 28 patients (20 males and 8 females) who had been followed up for 12 months were included. Their mean age at surgery was 61.2 ± 10.8 years and the average symptom duration was 23.36 ± 22.11 months. The gray values of circles 1, 2 and 3 were recorded as Gcompression, Gnorml and Gsac, respectively. Circle 1 was drawn with the maximum brightness point within the spinal cord as the center, circle 2 with the same area was plotted on the spinal cord with uniform echogenicity, without compression and at least 1 cm away from the circle 1, and circle 3 was drawn on the dorsal dural sac at the same segment as circle 1. GVR was calculated as follows: GVR-A = G_compression_/G_norml_ (method A), and GVR-B = G_compression_/G_sac_ (method B). The in-observer and inter-observer reliabilities of the two methods were compared. It is generally believed a reliability coefficient < 0.40 and > 0.75 indicate poor and good reliability respectively. The images-based GVR-B using this protocol demonstrates higher inter- and intraobserver reliabilities than GVR-A, and can be used as the basis for prognostic prediction and future studies.

**Results:**

All examination acquisitions were successfully completed. GVR-A averaged 2.043 (0.318–5.56), and GVR-B averaged 0.578(0.06–1.41). GVR-B has better repeatability of gray value measurement, smaller relative standard deviation (RSD%) (0.298 vs. 0.32) and larger inter-group correlation coefficient compared with GVR-A. The mean value (MD) of the GVR difference calculated by GVR-B between the two clinicians was closer to 0.

**Conclusions:**

For DCM patients routinely using ultrasound for real-time cord visualization during spinal cord decompression by French-door laminoplasty, the images-based GVR-B using this protocol demonstrates better inter- and intraobserver reliabilities compared with GVR-A.

## Introduction

Degenerative cervical myelopathy (DCM) is the most common non-traumatic disorder leading to neurological dysfunction in adults [1]. For DCM, the major pathological alterations of the spinal cord include parenchymatous degeneration and cystic necrosis caused by chronic spinal cord compression [2]. These two pathological alterations may lead to different neurological recoveries of DCM, but are always reflected as increased signal intensity (ISI) on T2W magnetic resonance imaging (MRI) [3]. T2W hyperintensity may also reflect transient or permanent microstructural changes such as edema, gliosis, inflammation, demyelination, spongiform changes, necrosis, and cavitation. Conventional MRI has been studied as a tool to assess the severity of spinal cord damage that has occurred, both in terms of correlation with current neurological status and prediction of post-surgical outcomes [4, 5]. The T2W signal intensity of the spinal cord is widely used to assess the impairment status and predict the neurological recovery of DCM [6]. However, conventional MRI consisting of T1w and T2w anatomical images offer only limited insight into pathological tissue changes, prompting the development of more advanced MRI sequences and emerging methods of imaging and electrophysiology that assess specific features of microstructure and tissue injury for the management of DCM [4, 5]. Recently, intraoperative ultrasonography (IOUS) was performed to evaluate and guide surgical decompression, and some IOUS-derived metrics were confirmed to be significantly correlated with the neurological function in DCM [7, 8]. Like ISI on T2W MRI, the spinal cord can manifest as hyperechogenicity on IOUS, with the same segment of ISI on T2W MRI [9–11]. The intensity of spinal cord hyperechogenicity is considered as a potential predictive indicator of neurological recovery in DCM after decompression [10]. To avoid the deviations caused by variations among machines, operators or observers, the gray value ratio (GVR) was measured to represent the intensity of spinal cord hyperechogenicity, by referring to the methods of measuring the signal change rate of the spinal cord on T2W MRI [11, 12]. Previously, two methods of measuring GVR with different reference elements were used to analyze the correlation between GVR and postoperative neurological recovery [9, 10, 13]. However, the accuracy of GVR which highly influences the accuracy of study conclusions may be subjected to many factors, especially the selections of region of interest (ROI) that differ among observers and among assessing timings of the same observer. Herein, we compared intra- and inter-observer reliabilities of two methods in evaluating the intraoperative ultrasonography based spinal cord hyperechogenicity intensity in DCM, aiming to find out a more reliable method. The results originating from this method are expected to be the basis of further studies.

## Materials and methods

### Study population

This study was approved by the Institutional Review Board of the studied hospital. Signed informed consents were obtained from all participants after explanation of the conductors.

A total of 33 consecutive patients with multilevel DCM (≥ 3) were prospectively enrolled between October 2018 and September 2019. Patients with a history of other spinal disorders, neurological injury, infection, tumor, or rheumatoid arthritis were excluded. Finally, 28 patients (20 males and 8 females) who had been followed up for 12 months were included. Their mean age at surgery was 60.8 ± 10.3 years and the average symptom duration was 40.7 ± 34.1 months (Table 1).

**Table 1:** Demographic data of patients

indicator	result
number of case	28
gender (male/female)	20/8
age at surgery (years)	61.2 ± 10.8
symptom duration (months)	23.36 ± 22.11

### Image acquisition

IOUS images were collected by the same surgeon in performing French-door laminoplasty according to Kurokawa’s method with some modifications [14]. After the detachment of bilateral paravertebral muscles, the centers of spinous processes were cut using a self-create and patented fretsaw. Bilateral gutters were created as hinges at the border of the laminae and facets. After the halves of the laminae were elevated and fixed to bilateral skin provisionally, normal saline was infused to form an acoustic window, then a linear array transducer of IOUS (M9Expert; Mindray Medical International Limited, Shenzhen, China) was used to observe the spinal cord and record the images [7, 9]. If residual compression was observed, further decompression under IOUS guidance was done. After observation, the appropriately- sized hydroxyapatite spacers were tied in place to bridge the bilateral edges of the laminae and were fixed with wires. Finally, a drainage tube was placed and the wound was closed in layers.

### IOUS measurements

The measurement protocol was formulated according to the experimental purposes above. The gray value of each ROI was measured by ImageJ (National Institutes of Health, Bethesda, MD, USA). All midsagittal images of the spinal cord (midsagittal slice was determined by visualizing the central echo complex of spinal cord) used to measure the GVR of hyperechogenicity were selected independently by the same two experienced surgeons who took part in the surgery. The GVRs of the two methods were measured independently by the same two researchers (a spine surgeon and a sonographer) and repeated three times (the interval between two measurements was not less than 7 days), following the same routine and using the same computer and software.

**Fig.1:**
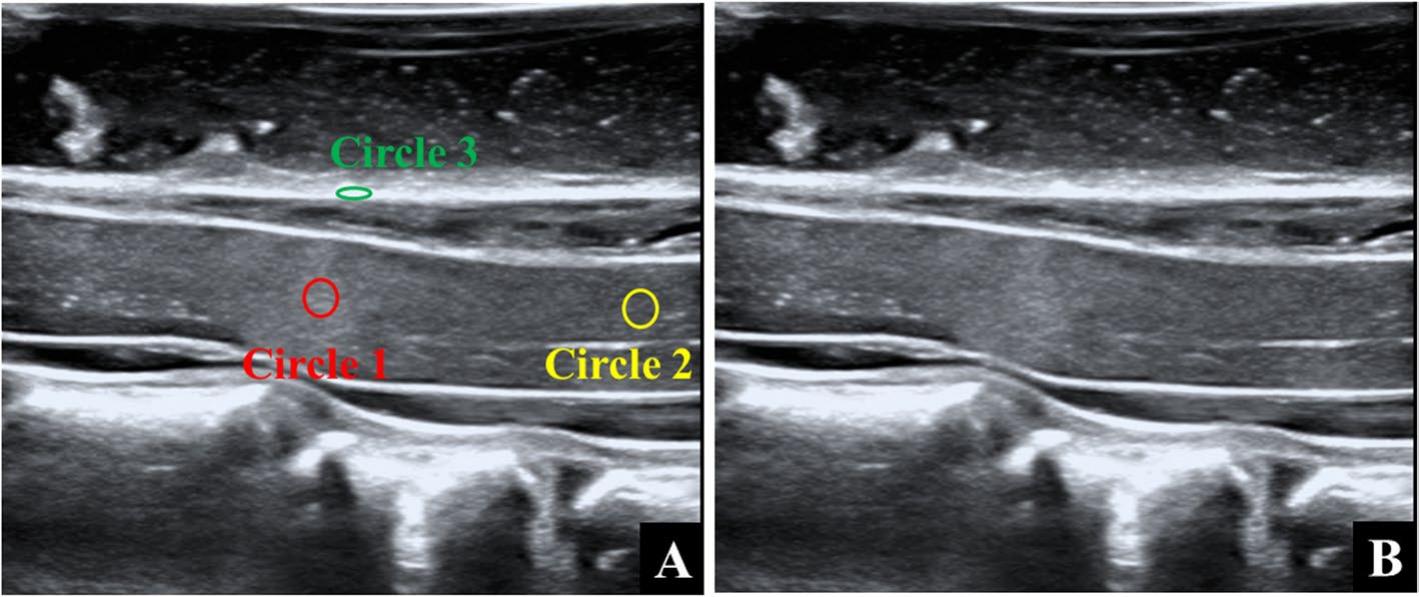
Measurements of echogenicity intensity: (**a**). Circle 1 (red) indicates hyperechogenicity intensity at the site of maximum compression level. Circle 2 (yellow) indicates echogenicity intensity at the site of compression-free level. Circle 3 (green) indicates echogenicity intensity of dural sac. (**b**). Original image of (**a**) without marks

For patients with macroscopic hyperechogenicity on IOUS, circle 1 was drawn with the maximum brightness point within the spinal cord as the center, circle 2 with the same area was plotted on the spinal cord with uniform echogenicity, without compression and at least 1 cm away from the circle 1, and circle 3 was drawn on the dorsal dural sac at the same segment as circle 1. For patients without different echogenicity within the spinal cord, circle 1 was drawn within the spinal cord at the most compressed level, and circles 2 and 3 were plotted according to the methods described previously. Importantly, the central canal of the spinal cord must be avoided in the drawing of circles 1 and 2. The gray values of circles 1, 2 and 3 were recorded as G_compression_, G_norml_ and G_sac_, respectively (Fig. 1A and B). GVR was calculated as follows: GVR-A = G_compression_/G_norml_ (method A), and GVR-B = G_compression_/G_sac_ (method B).

### Statistical analysis

Statistical analysis was carried out with IBM SPSS statistical software version 22.0 (IBM Corp., Armonk, NY, USA). Data were expressed as mean ± standard deviation (SD). The intra- and interobserver reliabilities of the gray value parameter measurements were quantified using the intraclass correlation coefficient (ICC), with a confidence interval of 95%. ICC, the full name is intraclass correlation coefficient, is one of the important reliability coefficient indicators for measuring and evaluating inter-observer reliability and test–retest reliability. ICC of 0.00—0.20, 0.21—0.40, 0.41—0.60, 0.61—0.80, and 0.81—1.00 is considered slight, fair, moderate, substantial, and almost perfect agreement respectively [15, 16]. It is generally believed that a reliability coefficient < 0.40 and > 0.75 indicates poor and good reliability respectively. For quantitative data, higher ICC is often required. The means of GVR were compared by T-TEST PAIRS. *P* values less than 0.05 were considered significant. To further acquire and illustrate a calculating method of GVR with higher reliability, we also adopted Bland–Altman analysis to account for the said results.

## Results

According to the formula to calculate the GVR, The GVR-A averaged 2.043 (0.318–5.56), GVR-B averaged 0.578 (0.06–1.41) after surgery.

### Ultrasonic signal parameters

Based on the calculating methods of GVR, Gnorml and Gsac were recorded three times at least one week apart. For the gray value, the intra-observer reliability was classified at different corresponding observation areas (Table 2).

**Table 2:** Comparison of results of gray value measured by observer A and observer B

method	n	Observer A ICC (95%CI)	Observer B ICC (95%CI)
*G*_*compression*_	28	0.85 (0.741–0.921)	0.979 (0.962–0.99)
*G*_*norml*_	28	0.826 (0.706–0.908)	0.922 (0.86–0.96)
*G*_*sac*_	28	0.794 (0.652–0.891)	0.874 (0.781–0.934)

*CI* confidence interval, *ICC* intraclass confidence correlation

At the maximum brightness point within the spinal cord, the intra-observer reliability of G_compression_ was classified as ‘‘almost perfect agreement’’ (Observer A ICC (95% CI): ICC0.85, ICC = 0.741—0.921; Observer B ICC (95%CI): ICC 0.979, ICC = 0.962—0.99, both *P* < 0.001) (Table 2).

At the spinal cord with uniform echogenicity, the intra-observer reliability of Gnorml was also classified as ‘‘almost perfect agreement’’ (Observer A ICC (95%CI): ICC0.826, ICC = 0.706 -0.908; Observer B ICC (95% CI): ICC0.922, ICC = 0.86 -0.96, both *P* < 0.001) (Table 2).

On the dorsal dural sac at the same segment with circle 1, and the maximum brightness point within the spinal cord, the intra-observer reliability of G_sac_ from Observer A resulted in ‘‘substantial agreement’’ (ICC (95%CI): ICC0.794, ICC = 0.652—0.891, *P* < 0.001), and the reliability coefficient still exceeds 0.75, indicating good reliability. Moreover, the intra-observer reliability of Observer B resulted in ‘‘almost perfect agreement’’ (ICC (95%CI): ICC 0.874, ICC = 0.781 – 0.934, *P* < 0.001) (Table 2).

### Intraobserver reliability

For GVR-A, the intraobserver reliability was classified as ‘‘moderate agreement’’ (Observer A ICC (95% CI):ICC 0.596, ICC = 0.391—0.767; Observer B ICC(95%CI): ICC0.595, ICC = 0.387—0.767, both *P* < 0.001) (Table 3).

**Table 3:** Intraobserver reliability of *GVR-A and GVR-B*

method	n	Observer A ICC (95%CI)	Observer B ICC (95%CI)
*GVR-A*	28	0.596 (0.391–0.767)	0.595 (0.387–0.767)
*GVR-B*	28	0.752 (0.594–0.866)	0.89 (0.807–0.943)

*GVR-A G*_*compression*_*/G*_*norml*,_ *GVR-B G*_*comp*_*ression/Gsac, CI* confidence interval, *ICC* intraclass confidence correlation

For GVR-B, the intraobserver reliability was good (Observer A ICC (95% CI):ICC 0.752, ICC = 0.594—0.866; Observer B ICC(95%CI): ICC0.89, ICC = 0.807—0.943, both *P* < 0.001) (Table 3).

### Interobserver reliability

For GVR-A, interobserver reliability was classified as ‘‘slight agreement’’ (ICC (95%CI): ICC0.198, ICC = -0.183—0.527, *P* < 0.001) (Table 4).

**Table 4:** Interobserver Reliability of *GVR-A and GVR-B*

method	n	Interobserver Reliability ICC (95% CI)
*GVR-A*	28	0.198 (-0.183–0.527)
*GVR-B*	28	0.86 (0.72–0.933)

*GVR-A* G_compression_/G_norml_*, GVR-B* G_compression_/G_sac_; *CI* confidence interval, *ICC* intraclass confidence correlation

For GVR-B, interobserver reliability was classified as ‘‘substantial to almost perfect agreement’’ (ICC (95%CI): ICC0.86, ICC = 0.72—0.933, *P* < 0.001) (Table 4).

### Comparison of two methods in calculating *GVR*

**Fig.2:**
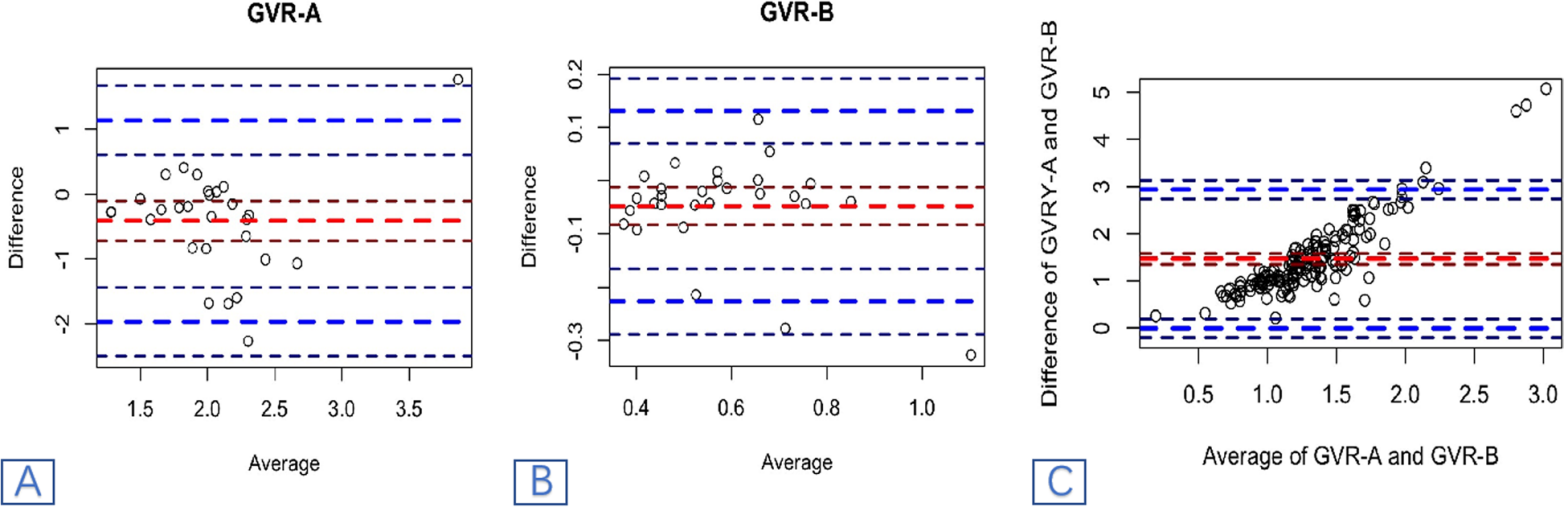
Comparison of two methods in calculating *GVR.*(**a**). Distribution results of GVR-A: x-axis and y-axis are the mean value and difference value of *GVR*-A, respectively;(**b**). Distribution results of GVR-B: x-axis and y-axis are the mean value and difference value of *GVR*-B, respectively;(**c**). Bland Altman analysis of results of GVR under two measurement methods: x-axis and y-axis are the mean value and difference value of *GVR*-A + *GVR*-B, respectively

According to the comparison of the two methods in calculating GVR (Table 5), the image-based GVR-B using this protocol has better repeatability, smaller RSD%, and larger ICC compared with GVR-A. The practical reliability significantly differs between the two methods (*P* < 0.05). The distribution results of GVR-A and GVR-B are shown in Fig. 2A and B.

**Table 5:** Comparison of two methods of calculating *GVR* (*x* ± *s*)

method	n	Observer A	Observer B	RSD%
*GVR-A*	28	1.837 + -0.671	2.25 + -0.577	0.32
*GVR-B*	28	0.555 + -0.157	0.602 + -0.186	0.298
t		10.298	-2.77	
p		0	0.01	

*GVR-A G*_*compression*_*/G*_*norml*_, *GVR-B G*_*compression*_*/G*_*sac*_

Furthermore, the two methods of measuring the GVR difference of the spinal cord were compared by Bland–Altman analysis (Table6, Fig. 2A, B and C). The mean value (MD) of the GVR difference calculated by GVR-B between the two clinicians was closer to 0 and smaller than that from GVR-A.It can be seen that compared with the GVR-A method, the method GVR-B has a higher consistency in measurement.

**Table 6:** Bland Altman analysis results of the *GVR* under two measurement methods

Items	*GVR* -A	*GVR*-B
MD	-0.413(-0.72—-0.106)	-0.048(-0.083—-0.0124)
lower limit of 95% LoA	1.14(0.608—1.671)	-0.227(-0.288—-0.165)
upper limit of 95% LoA	-1.966(-2.497—-1.434)	0.131(0.07—0.192)
t	-2.759	-2.77
P	0.01	0.01

*GVR-A:GVR-A G*_*compression*_*/G*_*norml*_, *GVR-A:GVR-B G*_*compression*_*/G*_*sac*_*;95% LoA:95% limits of agreement*

## Discussion

The gray values of different spinal cord elements including the maximal compressive level, compression-free level and the dural sac were measured, and the GVRs of maximal compressive level/compression free level and maximal compressive level/dural sac were calculated. The results of intra- and inter-observer reliability prove that the GVR of maximal compressive level/dural sac is more consistent for both inter- and intra-observer reliability.

In DCM, owing to the chronic compression, many pathological changes occur in the spinal cord, including ischemia, edema, neuron apoptosis, spinal cord atrophy and cystic necrosis [17]. Consistently, these pathological changes are always reflected as ISI on T2W MRI. This characteristic of MRI may cause the deviation when the signal intensity of T2W MRI ISI is used to evaluate the status and predict the neurological recovery of DCM. The parenchymatous degeneration and cystic necrosis indicate different severities of spinal cord impairment [18, 19]. The cystic necrosis of the spinal cord can also be reflected as T1W hypointensity, but the positive rate of T1W hypointensity is not high in DCM. This characteristic limits the application of T1W MRI in DCM [20].

The echogenicity of ultrasonography is based on the different densities of tissues [21]. For DCM, the chronic compression derived pathological changes may cause different densities of the spinal cord [22]. Then the different densities of the spinal cord are reflected as different levels of echogenicity on IOUS. Previously, we quantified the spinal cord hyperechogenicity as gray value, and revealed the negative correlation between hyperechogenicity intensity and postoperative neurological recovery [9, 10]. Besides, ultrasound has a significant image difference between liquid and parenchymal tissues, and thus can easily identify the pathology of cystic and parenchymatous changes. All those features of ultrasonography are unfulfilled on the clinical application of MRI or CT, and determine the application value and irreplaceability of IOUS.

According to the ultrasonography principles, differences in the density of adjacent tissues result in different echoes and are reflected as different gray values on ultrasound images [21]. The spinal cord hyperechogenicity in DCM patients results from the chronic compression subjected to the static and dynamic mechanical forces acting on the spinal cord [23, 24]. The chronic compression leads to fibrin deposition, and even fibrosis in the compression region, accompanied with the loss of nerve cells, proliferative fibroblasts and capillary endothelial cells [25, 26]. Eventually, the uneven density of the spinal cord will occur and appears as hyperechogenicity on IOUS [27, 28]. As shown in the typical case, spinal ultrasound can still detect signs that are not observed by MRI. When MRI presents only high signal changes, ultrasound can still return additional signs, such as syringomyelia signs. The intensity of spinal cord hyperechogenicity is considered as a potential predictor of neurological recovery in DCM after decompression. Therefore, the reliabilities of two methods to evaluate the hyperechoic intensity of DCM spinal ultrasound in the same observer and between observers were compared to find out a more reliable evaluation method.

The method used to calculate GVR by comparing the gray values of spinal cord hyperechogenisity and dural sac (GVR-B) is highly consistent for both inter- and intra-observer reliabilities, which are obviously higher than those of GVR-A. We attributed this improvement to the selection of ROIs, which, for intraoperative ultrasonic signal measurement, may be an important influence factor on GVR. G_compression_ (gray value of circle 1) has a one-to-one correspondence with Gsac (gray value of circle 3), but G_norml_ (gray value of circle 2) does not have such correspondence with G_sac_. The principle of ROI selection and the GVR-based calculation method reflect that G_norml_ is more random and highly variable, which also result in lower reliability of GVR-A. Conversely, when GVR-B was used to calculate the same G_compression_, more significant intra- and inter-observer reliability was obtained, indicating its consistency is better with the dura mater as a reference. Based on this finding, we suggest to use method B to evaluate the intensity of spinal cord hyperechogenicity in DCM.

There are several main limitations. First, as an exploratory prospective study, the sample size was relatively small. Second, no patient-based outcomes were evaluated.

Moreover, the lack of multicenter study can lower the reliability of our statistical analysis.

## Conclusions

The parameters of IOUS echogenicity measurement show high reliability both within and between observers. Our collective data support that the images-based GVR-B using this protocol has higher inter- and intra-observer reliabilities compared with GVR-A, and may be used as the basis for future studies.

## Data Availability

The datasets used and/or analyzed during the current study are available from the corresponding author on necessary and reasonable request.
