# Correction: Comparative intra- and inter-observer reliability of two methods for evaluating intraoperative ultrasonography-based spinal cord hyperechogenicity intensity in degenerative cervical myelopathy

**DOI:** 10.1186/s12891-022-05789-6

**Published:** 2022-09-06

**Authors:** Huachuan Wu, Guoliang Chen, Xianlong Li, Zhengya Zhu, Zuofeng Xu, Xizhe Liu, Shaoyu Liu

**Affiliations:** 1 Department of Orthopaedic Surgery, Innovation Platform of Regeneration and Repair of Spinal Cord and Nerve Injury, The Seventh Affiliated Hospital, Sun Yat-Sen University, Shenzhen, 518107 China; 2 Orthopaedic Research Institute/Department of Spinal Surgery, Guangdong Provincial Key Laboratory of Orthopedics and Traumatology, The First Affiliated Hospital of Sun Yat-Sen University, Guangzhou, 510080 China; 3 Department of Ultrasound, The Seventh Affiliated Hospital of Sun Yat-Sen University, Shenzhen, China

**Correction: BMC Musculoskelet Disord 23, 630 (2022)**

**https://doi.org/10.1186/s12891-022-05517-0**

Following the publication of the original article [1] the authors reported an error in the 2nd paragraph of section “Study population”.

“Their mean age at surgery was 60.8 ± 10.3 years and the average symptom duration was 40.7 ± 34.1 months.”

Should be corrected to:

“Their mean age at surgery was 61.2 ± 10.8 years and the average symptom duration was 23.36 ± 22.11 months.”

The original article [1] has been updated.
